# The Promoter of Rv0560c Is Induced by Salicylate and Structurally-Related Compounds in *Mycobacterium tuberculosis*

**DOI:** 10.1371/journal.pone.0034471

**Published:** 2012-04-02

**Authors:** Dorothée L. Schuessler, Tanya Parish

**Affiliations:** Barts and the London School of Medicine and Dentistry, Queen Mary University of London, London, United Kingdom; University of Hyderabad, India

## Abstract

*Mycobacterium tuberculosis*, the causative agent of tuberculosis (TB), is a major global health threat. During infection, bacteria are believed to encounter adverse conditions such as iron depletion. Mycobacteria synthesize iron-sequestering mycobactins, which are essential for survival in the host, via the intermediate salicylate. Salicylate is a ubiquitous compound which is known to induce a mild antibiotic resistance phenotype. In *M. tuberculosis* salicylate highly induces the expression of Rv0560c, a putative methyltransferase. We identified and characterized the promoter and regulatory elements of Rv0560c. P_Rv0560c_ activity was highly inducible by salicylate in a dose-dependent manner. The induction kinetics of P_Rv0560c_ were slow, taking several days to reach maximal activity, which was sustained over several weeks. Promoter activity could also be induced by compounds structurally related to salicylate, such as aspirin or para-aminosalicylic acid, but not by benzoate, indicating that induction is specific to a structural motif. The −10 and −35 promoter elements were identified and residues involved in regulation of promoter activity were identified in close proximity to an inverted repeat spanning the −35 promoter element. We conclude that Rv0560c expression is controlled by a yet unknown repressor via a highly-inducible promoter.

## Introduction

Tuberculosis (TB) is a major global health threat. According to the WHO, TB causes nearly 2 million deaths each year, and one third of the world's population is believed to be latently infected [1]. TB can be caused by any member of the *Mycobacterium tuberculosis* complex, however, *M. tuberculosis* is the major causative agent of TB in humans [1]. Despite an increased amount of research effort in the past decade, many processes underlying *M. tuberculosis* physiology and pathogenesis are still poorly understood. *M. tuberculosis* is an intracellular pathogen, and during infection the mycobacteria are believed to be exposed to adverse conditions such as hypoxia, nitric oxide and iron starvation [2]–[5].

Iron is an indispensable component of many prokaryotic and eukaryotic enzymes. When bacteria encounter conditions of low iron, for example during macrophage infection, they produce iron-sequestering siderophores in order to maintain cellular functions [6], [7]. *M. tuberculosis* produces two types of siderophores (mycobactins), whose production is essential for infection and survival in macrophages [8]–[10]. Expression of the genes required for mycobactin synthesis is controlled by the regulator of iron homeostasis IdeR [11]–[13]. Mycobactin biosynthesis involves the conversion of isochorismate into salicylate by the enzyme MbtI [14]–[16]. As a result of this, mycobacteria accumulate salicylate under iron-depleted conditions [14], [17]–[19].

Given the natural accumulation of salicylate under conditions encountered by the bacteria during an infection, it is interesting to note exogenous salicylate is able to induce a multiple antibiotic resistant (“mar”) phenotype in *M. tuberculosis* [20]. This effect is also seen in both Gram negative and Gram positive bacteria, and is thought to be due to the induction of efflux pumps via transcriptional repressors such as MarR in *Escherichia coli* or CmeR in *Campylobacter jejuni* [21]–[24].

The mechanism of the salicylate-induced mar phenotype in *M. tuberculosis* is poorly understood. Salicylate exposure affects the expression of 58 genes, none of which are known efflux pumps, and results in a general reduction of transcriptional and translational activities, as well as changes in energy metabolism [25]. Interestingly, two genes, Rv0560c and Rv0559c, are upregulated to a degree much higher (30- and 8- fold respectively) than any other of the differentially expressed genes [25].

Rv0560c is a non-essential gene encoding a putative benzoquinone methyltransferase. There have been suggestions that Rv0560c plays a role related to the biosynthesis of the isoprenoid lipid menaquinone [26], [27]. The fact that this gene is not expressed during aerobic growth [26], but upregulated during hypoxia [28] and intraphagosomal growth in macrophages [12] is interesting, indicating that this gene might play a role during infection. The Rv0560c protein is also induced in response to para-aminosalicylic acid (PAS), naphthoquinones such as menadione, and plumbagin, as well as the peroxisome proliferator gemfibrozil, and its structural relatives fenofibrate and clofibrate [26], [27].

The range of conditions under which Rv0560c is induced and the huge extent of its induction in response to salicylate are intriguing. The aim of this study was to identify and characterise the promoter of Rv0560c to gain further insight into its expression. Here, we demonstrate for the first time that Rv0560c is expressed from a salicylate-inducible promoter (P_Rv0560c_) which is highly active. We investigated the induction kinetics of this promoter, which took several days to reach maximal activity and remained highly induced for several weeks. We show that P_Rv0560c_ is also induced by structural analogues of salicylate as well as fenofibrates and is mildly induced under conditions of low iron. The −10 and −35 promoter elements, as well as residues involved in its regulation were identified. Our results suggest that this regulatory control is likely to be mediated via a repressor. The data presented here reveal P_Rv0560c_ to be a promoter with a high level of induction after salicylate treatment.

## Results

### Rv0561c and Rv0560c are expressed from separate promoters

**Figure 1:**
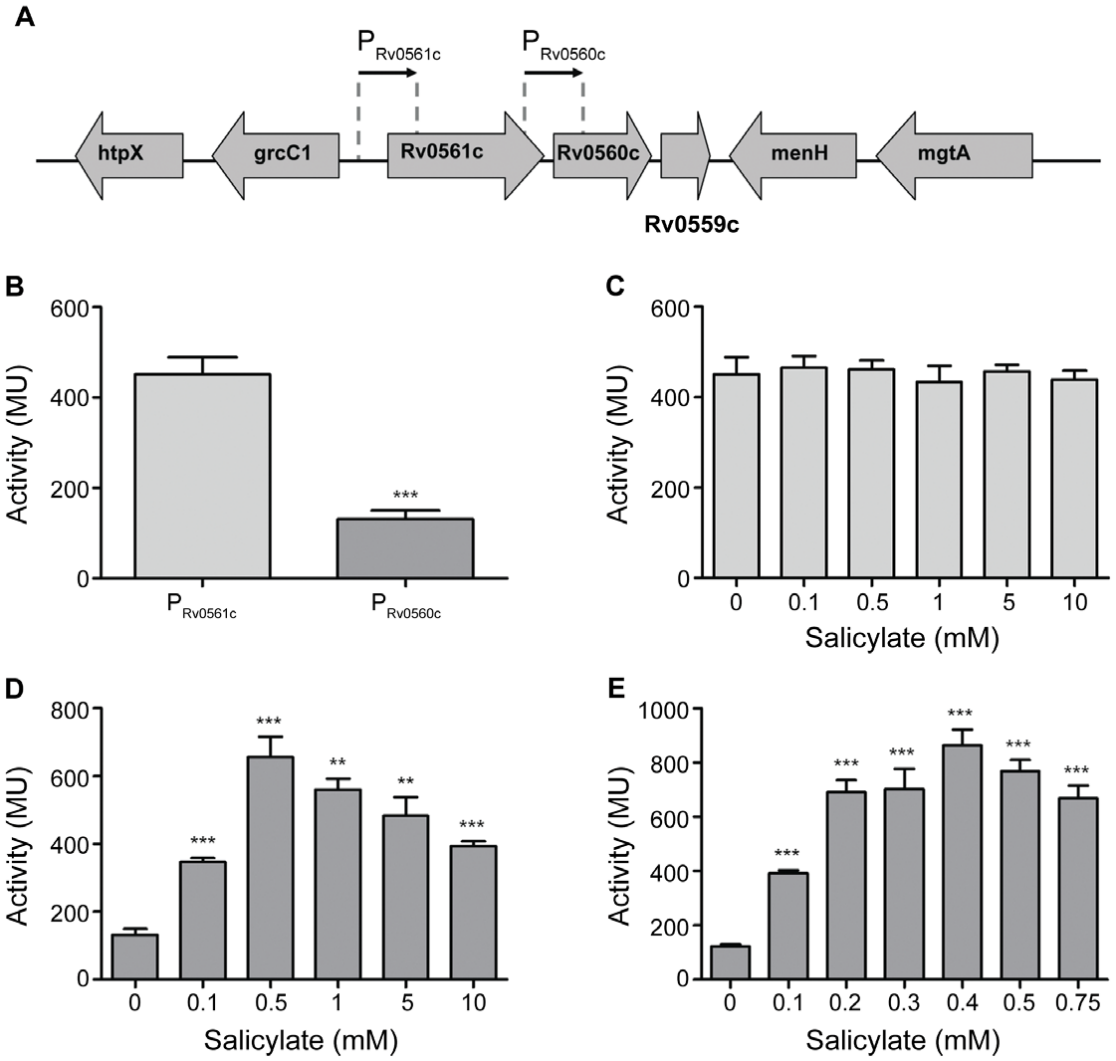
Identification of a salicylate-inducible promoter in *M. tuberculosis*. A. The genetic organization of Rv0561c and Rv0560c in *M. tuberculosis*. The regions tested for promoter activity are indicated as P_Rv0561c_, and P_Rv0560c_. B–E. Promoter activity was measured in *M. tuberculosis* transformants grown under aerobic growth conditions. B Activity in the absence of salicylate. C, D and E: Promoter activity of P_Rv0561c_ (C), P_Rv0560c_ (D and E), after 2 h treatment with varying concentrations of salicylate. Results are the average and standard deviation of three independent transformants assayed in duplicate. Activity is given in Miller Units. A significant difference compared using Student's t-test to the untreated control is marked by an * for p<0.05) ** for p<0.01, *** for p<0.0001.

We wanted to identify the promoter responsible for the salicylate-dependent induction of Rv0560c. Rv0560c has been suggested to be in an operon with its upstream gene Rv0561c and its downstream gene Rv0559c (Fig. 1A) [26]. Interestingly, the upstream gene Rv0561c has not been reported to be induced by salicylate [25].

To determine whether these genes had their own promoters, the upstream regions of both genes were tested for promoter activity in *M. tuberculosis* by linking them to a *lacZ* reporter gene. The upstream regions of Rv0560c and Rv0561c are referred to as P_Rv0560c_ and P_Rv0561c_ respectively (Fig. 1A). Promoter activity of ∼400 MU was detected from P_Rv0561c_, indicating that this upstream region contains an active promoter (Fig. 1B). Promoter activity was also detected from P_Rv0560c_ (130 MU) although lower than that of P_Rv0561c_ (Fig. 1B). Therefore each of the genes has its own promoter.

### P_Rv0560c_ is salicylate-inducible in M. tuberculosis

To test if one or both promoter regions were salicylate-inducible, we assayed the effect of varying concentrations of salicylate on promoter activity (Fig. 1C–E). P_Rv0561c_ activity did not change in response to treatment with salicylate (Fig. 1C) and thus, P_Rv0561c_ is not salicylate-inducible. In contrast to this, P_Rv0560c_ activity increased up to 6-fold to over 800 MU (Fig. 1D–E). Promoter activity did not increase any further with concentrations higher than 0.5 mM salicylate. Thus this promoter is the one responsible for the induction of Rv0560c upon salicylate exposure. P_Rv0560c_ is not only salicylate-inducible, but also displays dose responsive behavior at concentrations <0.4 mM (Fig. 1D–E), with maximal expression being achieved at 0.4 mM salicylate.

### P_Rv0560c_ induction kinetics

**Figure 2:**
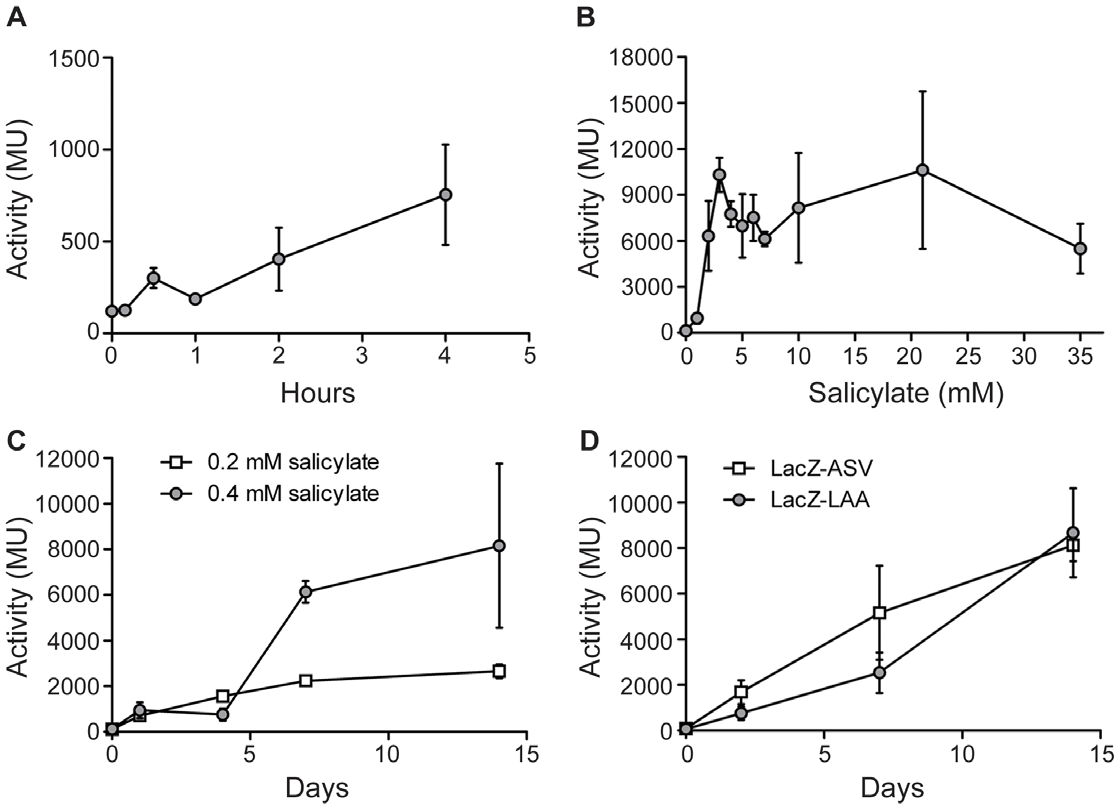
P_Rv0560c_ induction kinetics after exposure to salicylate in *M. tuberculosis*. A and B. Promoter activity was measured in *M. tuberculosis* transformants grown under aerobic growth conditions exposed to 0.4 mM salicylate (A and B), or 0.2 mM or 0.4 mM salicylate (C) or with the use of LacZ tagged for degradation(D). Results are the average and standard deviation of three independent transformants assayed in duplicate. Activity is given in Miller Units. LacZ-ASV was tagged with AANDENYAASV; LacZ-LAA was tagged with AANDENYALAA.

The induction kinetics of P_Rv0560c_ were investigated by monitoring promoter activity over time after treatment with salicylate. P_Rv0560c_ activity tripled from basal level to an activity of 301 MU after as little as 30 min of treatment with the inducer (p<0.05) and continued to increase over 4 hours (Fig. 2A). This trend persisted over the course of several days; a 100-fold increase (10315 MU) was observed after 3 d of treatment (Fig. 2B) and the promoter remained induced for at least 35 d (Fig. 2B). The level of induction is very high, and although the promoter responds in less than an hour to inducer, the full extent of the response takes several days and lasts for at least 5 weeks.

Induction kinetics changed with the amount of inducer present (Fig. 2C). With lower amounts of inducer (0.2 mM), activity of the promoter was lower with only a 20-fold induction being observed; and that only after 7–14 d of treatment. This shows P_Rv0560c_ to have slower induction kinetics and to be of lower strength when exposed to less inducer. Thus P_Rv0560c_ activity varies depending on inducer concentration and length of treatment.

The slow induction kinetics in our system could be due to accumulation of LacZ in the cells which is not degraded and accumulates over time. However, LacZ has been used widely as a reporter of promoter activity including determination of kinetics making this seem unlikely [29]. In order to test this possibility we constructed an unstable variant of LacZ which incorporated a protein degradation tag previously shown to function in mycobacteria [30]. Two LacZ variants incorporating either AANDENYAASV or AANDENYALAA at the C-terminal end were engineered; the induction kinetics from P_Rv0560c_ was measured as before. The induction kinetics were slightly slower for both variants taking 14 d to reach maximal, but still reached the high levels seen with native LacZ (Fig. 2D). The maximal level of expression was also slightly lower, 8,000 Mu compared to 10,000, but this likely reflects a higher turnover of synthesized protein carrying the degradation tag rather than reduced expression levels. Since the steady state levels were similar for all, we discounted the possibility that LacZ stability was responsible for increased activity over time.

### P_Rv0560c_ can be induced by structural analogues of salicylate

PAS, an antimycobacterial drug, and aspirin, a common painkiller, are structural analogues of salicylate; these compounds, as well as benzoate and the structurally-related compounds menadione, fenofibrate and gemfibrozil, have been shown to induce Rv0560c protein expression [26], [27]. We were interested in the effect of these compounds on P_Rv0560c_ activity.

**Figure 3:**
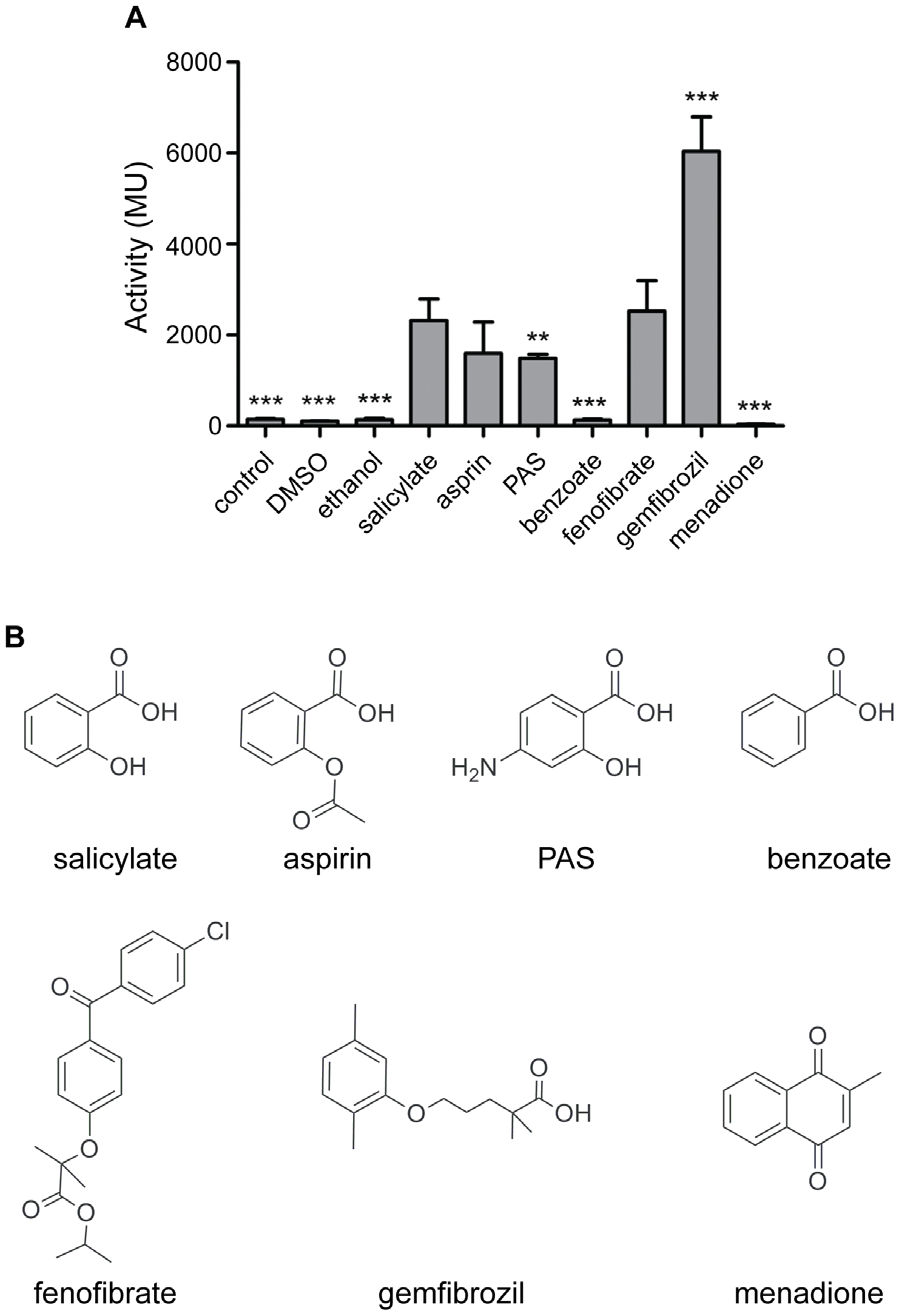
P_Rv0560c_ induction by structural analogs of salicylate in *M. tuberculosis*. Promoter activity was measured in *M. tuberculosis* transformants grown under aerobic growth conditions. A. Promoter activity of P_Rv0560c_ after treatment with 0.4 mM of compound for 3 d. Results are the average and standard deviation of three independent transformants assayed in duplicate. Activity is given in Miller Units. B. Chemical structures of compounds of interest. A significant difference compared using Student's t-test to the untreated control is marked by an * for p<0.05) ** for p<0.01, *** for p<0.0001.

We determined the effect of each compound on promoter activity at a fixed concentration (0.4 mM) (Fig. 3A). Salicylate resulted in a 64-fold induction (∼10000 MU), whereas its structural analogues aspirin and PAS induced a comparatively moderate 10-fold increase in P_Rv0560c_ (∼1500 MU; Fig. 3A). Surprisingly, the structural analogue benzoate did not induce P_Rv0560c_ activity at all (Fig. 3A). These results show that P_Rv0560c_ induction is specific to a certain structure present in salicylate, and to a certain extent in aspirin and PAS, but not benzoate (Fig. 3B).

Amongst the fibrates tested, fenofibrate evoked a 17-fold induction (2524 MU; Fig. 3A), and gemfibrozil evoked a 41-fold induction (6038 MU), which is closer to the levels achieved by salicylate exposure (Fig. 3B). Under the conditions tested here, menadione repressed P_Rv0560c_ activity 4-fold. These results show that other compounds that are not direct structural analogues of salicylate, are able to modulate P_Rv0560c_ activity.

### P_Rv0560c_ activity remains high after removal of exogenous inducer

We determined the off kinetics of the promoter. P_Rv0560c_ activity was induced to maximal level by growing *M. tuberculosis* in the presence of salicylate and promoter activity was monitored after removal of salicylate from the growth medium by washing in salicylate-free medium.

**Figure 4:**
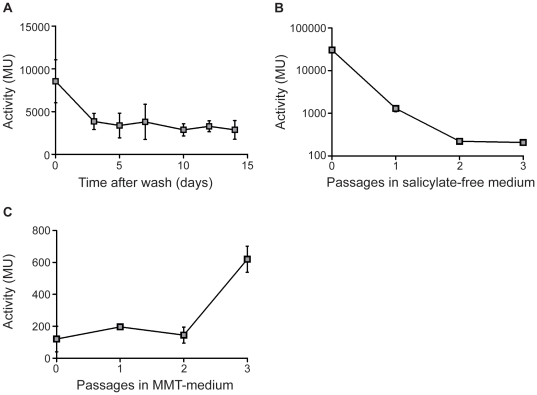
P_Rv0560c_ off kinetics in salicylate-free or iron-free medium in *M. tuberculosis*. *M. tuberculosis* transformants were grown under aerobic growth conditions in the presence of 0.4 mM salicylate. Cultures were washed and inoculated into salicylate-free medium A. Washed cells. Transformants were washed and resuspended in salicylate-free medium. B. Cells were washed and inoculated into salicylate-free medium; cultures were passaged into fresh salicylate-free medium at a dilution of 1/10. C. Cells were washed and inoculated in low iron minimal medium (MMT); cultures were passaged into fresh MMT medium at a dilution of 1/100. Results are the average and standard deviation of three independent transformants assayed in duplicate. Activity is given in Miller Units.

P_Rv0560c_ activity remained high immediately after the wash in salicylate-free medium (Fig. 4A), but then decreased over 3 d by approximately 2.5-fold (10000 to 3849 MU). Even after 2 weeks, activity did not decrease further, and was still 20-fold higher than the basal level of activity (∼150 MU). This shows that P_Rv0560c_ has slow off kinetics and that the remaining promoter activity could be due to the presence of residual salicylate in the medium. Increasing the number of washes carried out to remove salicylate from the growth medium up to four times did not allow for promoter activity to return to basal level over the course of 1 week (data not shown). Therefore, residual activity of P_Rv0560c_ could be due to high intracellular levels of salicylate which would not be removed merely by washing cells. To test this possibility, P_Rv0560c_ off kinetics were monitored during growth in salicylate-free medium during several passages; a salicylate-exposed culture was used to inoculate salicylate-free medium and cultured to late log phase before further passaging in salicylate-free medium. Promoter activity decreased with each passage (Fig. 4B), returning to basal level after 3 passages, suggesting that residual salicylate was gradually being consumed in the cells during growth.

### P_Rv0560c_ is induced in iron-depleted M. tuberculosis

Salicylate is known to accumulate in iron-depleted mycobacteria as an intermediate during the biosynthesis of the iron-sequestering mycobactin [14], [18], [19]. Hence, one would expect Rv0560c to be upregulated under this condition. However, existing data on whether Rv0560c is upregulated under iron-limiting conditions are conflicting [11], [26]. We tested if P_Rv0560c_ activity increased in *M. tuberculosis* grown in iron-free medium. After a state of iron-depletion had been achieved (three passages in iron-free medium), P_Rv0560c_ activity increased by 3-fold to 620 MU (Fig. 4C). Thus, as expected the promoter is induced when intracellular levels of iron are depleted. Interestingly induction was not as pronounced as in salicylate-exposed cells, suggesting that the normal intracellular concentrations of salicylate are low.

### P_Rv0560c_ is a negatively regulated, sigA-dependent promoter

**Figure 5:**
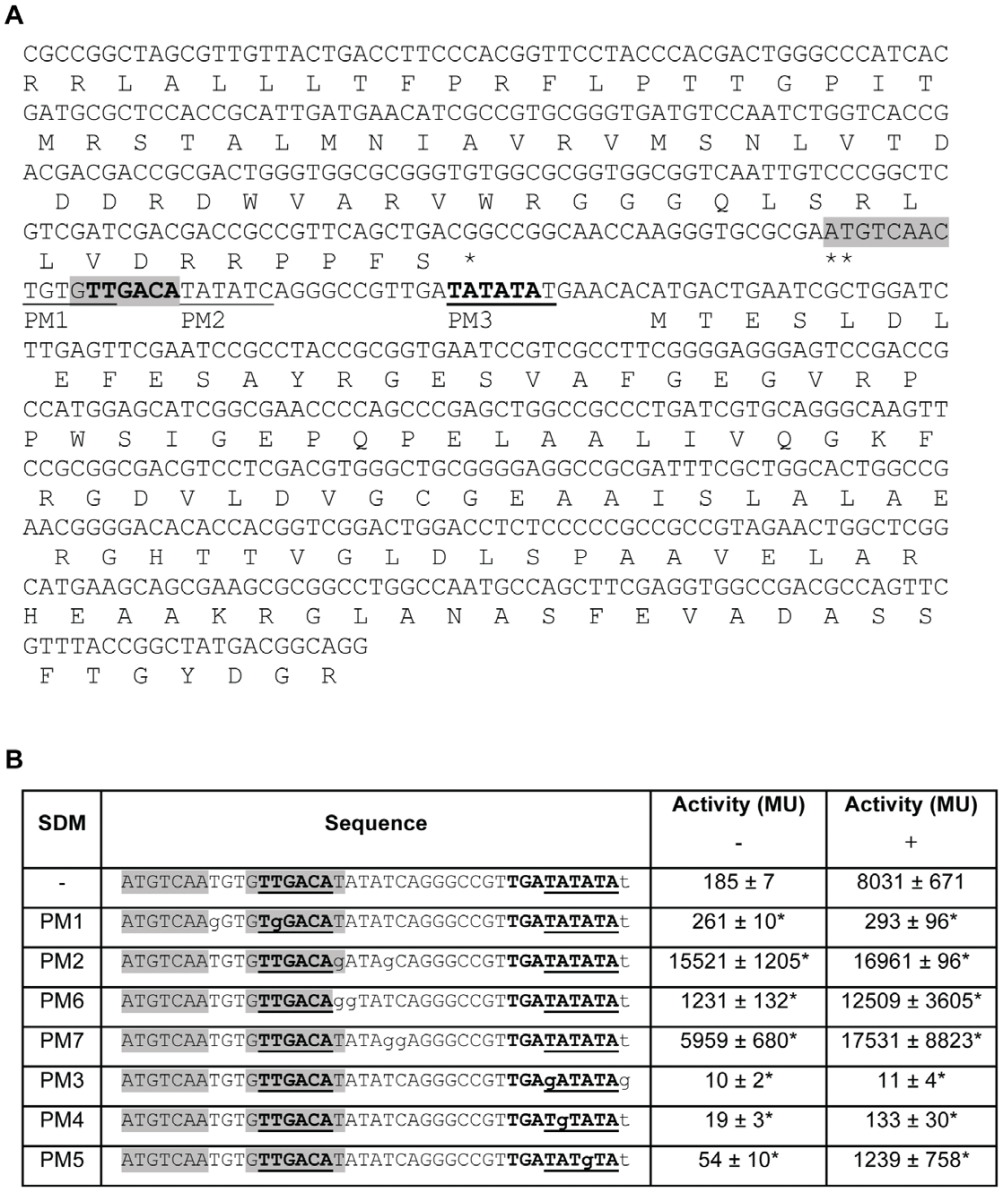
Identification of the promoter and regulatory elements. A. DNA sequence of the P_Rv0560c_ region. The predicted translation start site of Rv0560c according to TubercuList is marked with **. Protein sequences of Rv0561c and Rv0560c are shown. Potential −10 promoter elements (PM1, PM2, PM3) are underlined. The −35 and extended −10 element are in bold. A palindromic moitif is indicated by grey shading. B. Promoter activity following mutation of the promoter region. *M. tuberculosis* transformants were grown under aerobic growth conditions in the absence/presence of 0.4 mM salicylate. Results are the average and standard deviation of three independent transformants assayed in duplicate. Activity is given in Miller Units. A significant difference compared to the wild type is marked by an * (p<0.05).

To characterise P_Rv0560c_ and its regulator(s) further, an attempt was made to map its promoter elements. According to the current annotation on TubercuList (http://tuberculist.epfl.ch/), there is a short intergenic region of 24 bp between the stop codon of Rv0561c and the start codon of Rv0560c. A search for putative −10 elements within the upstream region of Rv0560c was carried out. Three putative −10 elements (PM1: TGTGTT, PM2: TATATC, PM3: TATATAT) were found, all located downstream of the annotated translational start site of Rv0560c, but immediately upstream of a putative alternative translational start site (Fig. 5A). To establish if and which one of these putative −10 elements was part of the Rv0560c promoter, residues in each motif were mutated.

Mutation of PM3 abolished promoter activity to a level seen in the empty vector control (11 MU) confirming that it is the most probable −10 element of the Rv0560c promoter. Interestingly, mutations in PM1 and PM2 both resulted in constitutive expression, albeit to different levels (Fig. 5B). Mutation of PM1 had minimal impact on the basal activity (260 MU), but resulted in complete lack of induction by salicylate (Fig. 5B). In contrast, mutation of PM2 resulted in high level constitutive activity (15000–17000 MU). Closer examination of the sequence revealed a putative −35 region with the consensus sequence TTGACA located 17 bp upstream of the confirmed −10 element; this region was mutated in PM1 (to TGGACA) suggesting that it is the −35 element and is involved in regulation of promoter activity.

The sequence of the promoter elements suggests that P_Rv0560c_ is highly likely to be a SigA–dependent promoter. The SigA consensus sequence is TTGACW-N_16–21_-TATAMT [31]. The promoter region identified has a perfect match in the −35 region of TTGACA, with a spacing of 17 bp to the −10 element TATAta (matching bases in capital). Furthermore, P_Rv0560c_ looks to be an extended promoter due to the presence of a TGN motif (in this case TGA) directly in front of the −10 element [32].

### Mutations in the −10 element affect promoter strength

We decided to mutate some of the residues in the −10 element to test their effect on promoter strength and regulation (Fig. 5B). Mutation of the second residue (PM4) severely weakened promoter strength. Basal activity under uninduced conditions was abolished. However, the promoter retained inducibility, albeit to a lower level (133 MU). Similar effects were seen when mutating the fourth residue (PM5), although the promoter was slightly more active (54 MU and 1239 MU in the uninduced and induced states respectively). These results are as expected since mutating residues away from the sigma-factor consensus would weaken recognition and binding of the factor, resulting in less transcription.

### Identification of further residues involved in repressor binding

Since mutation in the −35 region resulted in loss of induction, we predicted that there is a regulatory element in this region; such operator regions are often located between the −35 and 10 elements. We carried out further mutations to identify other residues involved in regulation of promoter activity located between the −10 and −35 region. Mutation of the two residues immediately downstream of the −35 element (PM6) resulted in a much higher basal level of activity (1232 MU). Interestingly, mutation of two bases further downstream (PM7) increased the basal activity even further (5959 MU). In both cases, induction was retained, with promoter activity of over 10000 MU in the presence of salicylate (Fig. 5B). This confirms that this region contains a regulatory site and suggests it is the binding site for a repressor.

## Discussion

We were interested in the *M. tuberculosis* gene Rv0560c due to its strikingly high induction in response to salicylate, and its upregulation under conditions mimicking the *in vivo* environment the bacteria encounter during an infection [12], [28]. Although Rv0560c has been suggested to be in an operon with its upstream gene, our results show that each of the genes does have its own promoter, both of which are active during aerobic growth. Furthermore, we demonstrate that P_Rv0560c_, but not P_Rv0561c_ is inducible by salicylate. This is in accordance with a previous transcriptome study showing that Rv0560c, but not Rv0561c is upregulated after salicylate treatment [25].

We found P_Rv0560c_ to be a strong promoter as compared to other *M. tuberculosis* promoters and with slow induction kinetics, taking several days to reach peak activity and remaining stably induced over a course of several weeks. The majority of *M. tuberculosis* promoters measured using LacZ as a reporter have activity in the range of 100–1000 MU; examples include the promoters of *recA*, *pknH*, *embA*, *mbtB* and *higBA* [33]–[37]. There are few examples of promoters with activity in the 10,000 range; for example P_rpfA_, which is reported as one of the strongest constitutive promoters in *M. tuberculosis* has an activity of 4500 MU [38], [39], whereas P_whiB1_ has comparable activity to P_Rv0560c_ at 800–15,000 MU [40].

The high level of promoter activity after induction could be due to presence of a perfect match to the canonical −35 element TTGACA (which is not always present in most mycobacterial promoters), the extended promoter motif, and the high sequence similarity of the −10 element to the consensus sequence TATAAT [31], [32]. Indeed, these attributes are also present in the P_whiB1_ promoter with comparable activity [41].

The slow induction kinetics could be due to a tight interaction of the repressor with its binding motif, taking a long time to alleviate and allow full access to the polymerase. Alternatively the mechanism leading to alleviation of repression (whether it occurs through salicylate binding to the repressor or via a relay of signal through other regulators) might be slow. It is interesting to note that there are other inducible promoters with slow kinetics, although most of these are not native *M. tuberculosis* systems, for example the ATc and pristinamcyin systems [42]–[44]. However, the kinetics of induction of P_Rv0560c_ appear to be particularly slow, since the ATc-inducible systems are fully induced with 24 h [36], [43], [45], [46] and even the “slow” induction of RecA expression previously noted in *M. tuberculosis* took 18–36 h, rather than 72 h [33]. These kinetics are not a general phenomenon since other promoters can be induced to maximal expression much more rapidly, for example induction of heat shock proteins takes less than an hour.

The fact that some structural analogues of salicylate (PAS and aspirin), but not others (benzoate) induce promoter activity confirms that induction is specific to a certain chemical structure present in salicylate, PAS and aspirin, but not benzoate. These findings are in accordance with a previous study on Rv0560crotein expression, except for aspirin (of which salicylate is a breakdown product) which was reported not to induce Rv0560c [26]. 

Compounds that can interfere with isoprenoid quinone action and are structurally related to salicylate (such as fenofibrate or gemfibrozil) also induced P_Rv0560c_ activity. In our study, menadione did not induce P_Rv0560c_, but actually repressed it. Both results are in accordance with previous findings of a protein study [27]. It would be interesting to determine whether this induction is due to an indirect effect or due to a structural motif common to all these compounds.

The function of Rv0560c is unknown, but it has homology with a benzoquinone methyltransferase (UbiG) involved in ubiquinone biosynthesis in *E. coli* [26]. Quinones are lipid-soluble electron carriers involved in the electron transport chain, a process essential for growth. Rv0560c could be involved in a ubiquinone biosynthetic process, as *M. tuberculosis* does contain homologs of some of the genes present in the *E. coli* ubiquinone pathway [47], although there is currently no evidence that mycobacteria produce ubiquinones [48], [49].

Rv0560c has also been suggested to be involved in menaquinone biosynthesis, due to its genomic proximity to *menH* (Rv0558), *menD* (Rv0555) and *menC* (Rv0553) [26], [27]. Menaquinone biosynthesis is essential for mycobacterial viability and this synthetic pathway has been proposed as an attractive target for novel antimycobacterial drugs [50], [51]. Furthermore, a recent study linked menaquinones to the induction of the DosR regulon, which is implicated in the adaptation to hypoxia and the establishment of a dormant state [52], [53]. One could speculate Rv0560c is a methyltransferase carrying out functions equivalent of the methyltransferases MenH or MenG.

Alternatively, Rv0560c (which is not expressed during aerobic *in vitro* growth) could be involved in the synthesis of novel menaquinones such as the recently identified sulphated menaquinone [54] and/or menaquinone biosynthesis under certain stress conditions such as iron starvation. Our results show P_Rv0560c_ to be induced during iron starvation, when intracellular levels of salicylate are naturally elevated. The salicylate-dependent induction of P_Rv0560c_ accounts for the upregulation of Rv0560c during iron depletion, despite the absence of binding motifs for the main regulator of iron responsive genes (IdeR) upstream of Rv0560c [11], [13].

The aim of our study was to identify and characterize the promoter of Rv0560c. Our results demonstrated P_Rv0560c_ to be a predicted SigA-dependent promoter and suggest that the translational start site of Rv0560c is currently misannotated. Furthermore, the expression of Rv0560c appears to be regulated by a repressor, which according to our results binds to residues close to the −35 element of P_Rv0560c_, possibly to a palindromic motif that overlaps the −35 element. This is supported by the fact that when certain residues in this motif are mutated, P_Rv0560c_ is constitutively active at maximal level.

In Gram negative and Gram positive bacteria the salicylate-induced mar phenotype is mediated through salicylate binding directly to transcriptional repressors of the MarR family [21]–[23]. In *E. coli*, MarR binds to salicylate, relieves repression of its regulon and evokes induction of a mild antibiotic resistance phenotype through upregulation of efflux pumps [21]–[23], [55], [56]. The mar phenotype has been observed in *M. tuberculosis* although the mechanism of induction of multidrug resistance has not been determined [20]. Interestingly, *M. tuberculosis* possesses several MarR type regulators, with Rv1404 or Rv2887 showing the highest sequence similarity to the *E. coli* MarR, with some conservation of the salicylate binding sites. Thus either of these genes could make attractive candidates for the regulator of Rv0560c expression.

To conclude, we identified Rv0560c to have its own salicylate-inducible promoter. with a high level of induction. We present evidence that this promoter is controlled by an unknown repressor binding to a palindromic motif upstream of Rv0560c. Further studies are required to identify this repressor, which we speculate to be part of the MarR family. Whether Rv0560c is involved in menaquinone biosynthesis, if it is important for iron starvation; salicylate tolerance or even plays a role in the mar phenotype are interesting questions which would be worthy of further investigation.

## Materials and Methods

### Bacterial strains and culture conditions

*M. tuberculosis* H37Rv (ATCC 25618) was grown in Middlebrook 7H9 medium supplemented with 10% v/v oleic acid-albumin-dextrose-catalase (OADC) supplement and 0.05% w/v Tween 80, or on 7H10 agar supplemented with 10% v/v OADC. Cultures were grown without agitation in 50 mL conical tubes unless otherwise stated. Streptomycin was used at 20 mg L^−1^ and X-gal at 50 mg L^−1^ when required. Low iron minimal medium (MMT) was prepared as follows: 6 g L^−1^ Na_2_HPO_4_, 3 g L^−1^ KH_2_PO_4_, 0.5 g L^−1^ NaCl, 1 g L^−1^ NH_4_Cl and 0.0147 g L^−1^ CaCl_2_ supplemented with 0.05% w/v Tween 80 and 2% v/v glycerol and treated overnight with 5 g L^−1^ Chelex 100; 2 mM MgSO_4_ was added and the medium was filter-sterilised.

### Plasmid construction

Primers were designed to amplify the upstream regions of Rv0561c and Rv0560c from *M. tuberculosis* genomic DNA using primer pairs UR561F CCCCCCGGGGGATC- GCGACGTTGTTAC and UR561R CCCCCCGGGCCGCCAGCCACTTAC for Rv0561c, and UR560F CCCCCCGGGGCGCCGGCTAGCGTTGTTAC and UR560R CCCCCCGGGCCTG- CCGTCATAGCCGGTAAACG for Rv0560c. Products were cloned as *Sma*I fragments (underlined) into the *Sac*I site of pSM128 [57] upstream of the *lacZ* reporter gene. Protein tags were added to *lacZ* in pSM128 using SDM primer pairs TailLAAf GGTCTGGTGTCAAAAAGCAGCAAACGACGAAAACTACGCTTTAGC AGCTTAATAATAAC, TailLAAr GTTATTATTAAGCTGCTAAAGCGTAGTTTTCG TCGTTTGCTGCTTTTTGACACCAGAC or TailASVf GGTCTGGTGTCAAAAAGC AGCAAACGACGAAAACTACGCTGCATCAGTTTAATAATAAC, TailASVr GTTA TTATTAAACTGATGCAGCGTAGTTTTCGTCGTTTGCTGCTTTTTGACACCAGACC.

### Site directed mutagenesis (SDM)

Amplification reactions were carried out in 50 µL total volume containing 2.5 units *PfuUltra* Hot Start high fidelity DNA polymerase (Stratagene), 1× buffer, 0.5 mM dNTPs, 10 pmol of each primer, 5 µL DMSO, and 10 ng template. The thermocycling programme used was: 94°C for 2 min, followed by 18 cycles of 94°C for 30 s, 56°C for 1 min and 68°C for 9 min, followed by 68°C for 10 min. Template was degraded using 10 units *DpnI* (Promega) at 37° for 2 h. 10 µL of each reaction were used to transform competent *E. coli*. Recombinant plasmids were isolated and sequence-verified.

### Preparation of cell-free extracts for promoter activity assays

Electrocompetent mycobacteria were prepared as described previously [58], electroporated with 1 µg plasmid DNA and transformants selected on streptomycin. *M. tuberculosis* transformants were cultured to mid-log phase and exposed to compounds. Cells were harvested, washed and resuspended in 1 mL 10 mM Tris-Cl (pH 8) and added to 2 mL lysing matrix B tubes (MP Biomedicals) on ice. Cells were disrupted using a 30 s cycle at speed 6.0 using a FastPrep™ FP120 (MP Biomedicals). Extracts were centrifuged at 16000× g for 4 min and the supernatants recovered filter-sterilised through a 0.2 µm filter unit and recovered. Total protein concentration of the samples was determined using the BCA protein assay kit.

### Quantification of ß -galactosidase

Assays of ß-galactosidase activity were carried out as previously described [59]. To 100 µL of cell-free extract, 900 µL of Z-Buffer (60 mM Na_2_HPO_4_, 40 mM Na_2_PO_4_, 10 mM KCl, 1 mM MgSO_4_, pH 7) was added. Samples were pre-warmed to 37°C for 5 min and 200 µL of 4 mg mL^−1^ ONPG was added. Reaction mixtures were incubated at 37°C and reactions were stopped with 500 µL of 1 M NaHCO_3_ after 30–90 min. The OD_420_ was measured and ß-galactosidase activity was calculated as Miller units (MU) = amount of O-nitrophenol produced (nmol) per min per mg of total protein, using the following formula: Units = (OD_420_×1.7)/(time (min)×volume of cell-free extract (mL)×total protein concentration (mg mL^−1^)×0.0045).
